# Optimal Use of Advanced Practice Providers at an Academic Medical Center: A First-Year Retrospective Review

**DOI:** 10.7759/cureus.34475

**Published:** 2023-01-31

**Authors:** Vasco Deon Kidd, Alpesh Amin, Nitin Bhatia, Denise Healey, Courtney Fisher, Mojgan Rafiq, Mary Jo Angelica E. Gallegos, Kathrina Munoz

**Affiliations:** 1 Orthopaedic Surgery, University of California Irvine, Orange, USA; 2 Medicine, University of California Irvine, Orange, USA; 3 Clinical Affairs, University of California Irvine, Orange, USA; 4 Ambulatory Administration, University of California Irvine, Orange, USA; 5 Urology, University of California Irvine, Orange, USA

**Keywords:** physician productivity, hospital contribution margin, work relative value units, health policy, medical specialty, economics, academic medical center, nurse practitioner, physician associate, physician assistant

## Abstract

Background

Physician assistants/associates (PAs) and nurse practitioners (NPs), together known as advanced practice providers (APPs), practice with a high degree of clinical autonomy and professional respect, and play a critical role in team-based care. Aligning APP care delivery models to promote top-of-license practice is essential to improving ambulatory capacity and bottom-line expectations at academic medical centers (AMCs) in the 21^st^ century and beyond. This administrative quality improvement study assesses the downstream impact of restructuring our APP care models to promote independent practice sessions.

Methods

Our AMC formed an APP oversight committee in April 2021 to optimize the ambulatory care model, realign APP funds flow, and set performance standards to which PAs and NPs are being held accountable. We conducted a one-year retrospective review of internal data from July 2021 to June 30, 2022. Certified registered nurse anesthetists (CRNAs) were excluded from this analysis.

Results

APP productivity year-over-year (YOY) aggregate data across all School of Medicine (SOM) departments, demonstrated a 53% increase in work relative value units (wRVUs), 84% increase in payments, and 79% increase in charges from the prior fiscal year (July to June). Regarding APP ambulatory clinical effort (YOY), there was a 45% increase in the number of APP completed visits (92% return patient visits, 8% new visits). An increase in APP productivity did not adversely impact patient satisfaction, physician-generated wRVUs, or delay programmatic expansion efforts. Lastly, in a recent engagement survey, the majority of PA and NP respondents (78%) reported working either “most of the time” or “always” at the top-of-license.

Conclusion

This quality improvement study demonstrates that enhancement of PA and NP utilization through top-of-license initiatives can be achieved without jeopardizing physician wRVUs or performance. While we acknowledge, there are differences between healthcare institutions in terms of care delivery and compensation models, organizational culture, and distribution of clinical resources, there remains an opportunity among hospitals and health systems to optimize this critical and essential APP workforce.

## Introduction

Advanced practice providers (APPs) have been deeply immersed in the world of American medicine since the 1960s. The physician assistant/associate (PAs) profession grew 28.7% between 2017 and 2021, reaching 158,470 certified PAs at the end of 2021. Meanwhile, currently there are 355,000 nurse practitioners (NPs) licensed to practice an increase of 9% from the estimated 325,000 reported in May 2021 [1,2]. PAs are trained as generalists in the medical model while NPs are trained based on a specific population foci. Postgraduate training options in the form of residencies or fellowships exist but are not a requirement for PA and NP entry-level practice or state licensure [3,4]. PAs and NPs clinically practice in various areas of medicine and are integral to a highly effective team-based care model. It is well documented that PAs and NPs provide safe, cost-effective care with excellent patient outcomes [5,6]. Academic medical centers (AMCs), however, have struggled to fully utilize PAs and NPs at their fullest potential in large part due to poorly defined roles and responsibilities, misunderstanding of professional scopes of practice and training, and perception by some that PAs and NPs may be in competition with physicians for patient visits. As hospitals and health systems try to navigate turbulent financial and market challenges, it is important that all providers are working at top-of-license, which improves clinician satisfaction, quality of care, and financial outcomes [7].

Historical context and problem

University of California Irvine (UCI) Health is a 459-bed acute care hospital that includes tertiary and quaternary care services, ambulatory and specialty medical clinics, and behavioral health and rehabilitation services. UCI Medical Center features Orange County’s only National Cancer Institute-designated comprehensive cancer center, a high-risk perinatal/neonatal program, the county’s only regional burn center, and is an American College of Surgeons-verified Level I adult and Level II pediatric trauma center. Our large AMC employs approximately 220 APPs (NPs, PAs, and CRNAs) across inpatient and outpatient areas. Over the past two years, APP expenditures have increased exponentially without an incremental revenue offset. This financial imbalance led to concern among School of Medicine (SOM) leadership that APPs in some clinical areas were being significantly underutilized. In late 2019, the institution hired a director of advanced practice to provide guidance around role clarity, credentialing, compliance, scope of practice, and optimal utilization of APPs. This hiring coincided with the establishment of an office of advanced practice and development of outpatient and inpatient practice standards.

After the arrival of the director of advanced practice, a multidisciplinary engagement survey was developed and distributed to the advanced practice workforce to assess the current state of practice, including factors for improving satisfaction, retention, utilization, and alignment of APP clinical effort. Survey data along with evaluation of specific practice sites by the director of advanced practice, revealed that there was considerable variability in APP care delivery models, utilization, and performance expectations. Consequently, many APPs were performing below-license tasks such as obtaining authorizations, rooming and exiting patients, filling out disability paperwork, obtaining medical records, faxing forms,​ performing case management activities, and scribing for the supervising physician in paired shared clinics. Most, if not all, of these activities can be done by other members of the care team. Moreover, the ambulatory APP salary and benefit expense was largely borne at the enterprise level, creating a disincentive for SOM departments to optimize their APP workforce.

In January 2021, the AMC rehired an external consultant group to address barriers and provide guidance on a system-wide approach for implementation of practice standards across the enterprise. The ambulatory standards mainly focused on transitioning PAs and NPs into independent practice sessions. The aim of this administrative study was to evaluate the impact of implementing PA and NP independent practice sessions on ambulatory capacity, bottom-line expectations, patient satisfaction, and provider productivity. 

This article was previously posted to the Research Square preprint server on October 20, 2022 [8].

## Materials and methods

APP oversight committee composition

To address these local challenges and pivot our advance practice provider workforce to top-of-license practice, an APP oversight committee was formed. The committee was co-chaired by the director of advanced practice, chair of the medicine department, and assistant dean of workforce planning. The executive sponsors of the committee were the vice dean of clinical affairs and the chief operating officer (COO). The committee members were comprised of the chief nursing executive (CNE), medical director for oncology services, and chairs of the following departments: plastic surgery, orthopaedic surgery, anesthesia, neurology, dermatology, general surgery, and otolaryngology. The committee was tasked with enhancing existing ambulatory practice expectations, evaluating APP funds flow options, and monitoring the deployment of the APP practice standards enterprise-wide.

APP ambulatory and inpatient practice standards

After a comprehensive discussion and review of industry standards, the APP oversight committee elected to adopt a 36-hour patient-facing minimum for all ambulatory PAs and NPs [8]. Additionally, ambulatory PAs and NPs were to have their own individual clinic templates under their name as opposed to working off a physician’s template or general template, which renders the APP’s work contribution invisible. APP inpatient guiding principles included expectations based on a co-management strategy, average daily census (ADC) data, inpatient throughput goals, procedural volume, patient to APP ratios, and graduate medical education (GME) considerations. Additionally, APP performance monitoring is performed through our well-established Ongoing Professional Practice Evaluation (OPPE) process and newly approved APP performance evaluation tool.

APP funds model redesign

Prior to the redesign, salary/benefit expenses of the ambulatory PAs and NPs were either completely funded by the enterprise or partially funded through an offset of professional fee collections earned by the APP. Under the new funds flow methodology, the salary and benefit expenses associated with ambulatory PAs and NPs became the financial responsibility of each department where the APP was employed. Each department set a standard performance benchmark based on expected visit volume per session (four-hour block per session) along with a defined minimum work relative value units (wRVU) performance expectation required to generate sufficient funds to cover salary/benefit expenses and achieve breakeven. The number of expected sessions per year was based on an assumption of 44 work weeks per year x nine sessions per week adjusted to percent appointment. Salary deficits resulting from insufficient ambulatory visit volumes would require funding at the department level. Departments with PAs and NPs with dual responsibilities, i.e., both outpatient and inpatient activities, received enterprise funding in proportion to the percent of APP inpatient effort. The new APP fund flows methodology was agreed upon by the enterprise in May 2021 and operationalized in July 2021. Volumetric key performance indicators (KPIs) were established for PAs and NPs and were routinely monitored through a centralized dashboard developed by the director of advanced practice, analyst in the office of advanced practice (OAP), and decision support team [9].

Deployment strategy

**Figure 1:**
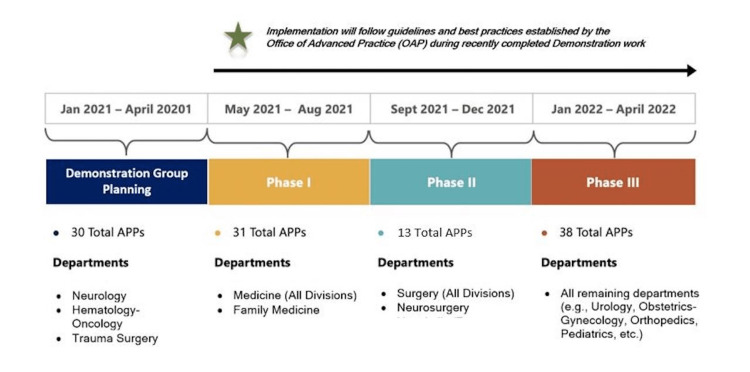
Implementation Roadmap APP: advanced practice provider

The director of advanced practice was responsible for deploying the new strategy using an implementation road map framework (Figure 1).

Prior to the rollout of the new ambulatory practice standards, a high-level assessment was completed by ambulatory directors’ team and the director of advanced practice on the anticipated level of support needed to transition ambulatory PAs and NPs into independent practice sessions (Table 1).

**Table 1:** Staffing and Clinic Volume Projections FY: fiscal year, APP: advanced practice provider

FY 21 APP Clinic Volume	28,042
Projected FY 22 APP fully independent clinic volume	69,080
Potential APP volume increase	41,038
% Potential APP volume increase	146.34%
FY 22 additional APP independent sessions	200
Potential ancillary full time Equivalents (FTEs) needed to support independent APP clinic sessions	58 FTEs

The organization started with an egalitarian view that PAs and NPs would require the same level of ancillary support for their independent sessions as faculty working in the same SOM specialty or subspecialty. After the assessment was completed, the director of advanced practice met with PAs and NPs, practice managers, faculty, and department administrators to discuss the new changes and implementation strategy. Department chairs (physician champions) also met with their faculty and APPs to reinforce the new ambulatory practice and performance expectations. The director of advanced practice was responsible for providing monthly updates to the oversight committee on the deployment of the ambulatory practice standards and other issues pertinent to the advanced practice provider program. 

## Results

APP productivity assessments were based on volume-based wRVUs derived solely from billable activities where the APP was the rendering or performing provider of record. One year after implementation of the ambulatory/inpatient practice standards, SOM aggregate APP productivity data demonstrated a 53% increase in wRVUs, an 83% increase in payments, and a 79% increase in charges from the prior fiscal year (July to June). The following departments saw the most significant improvement in APP productivity year-over-year (YOY) (Table 2). There was no adverse impact on faculty-generated wRVUs. Lastly, when comparing physician wRVUs (excluding APPs), overall physician wRVU productivity increased by 3% YOY from the below list of clinical departments.

**Table 2:** Productivity by Clinical Department FY: fiscal year, wRVU: work relative value units, YOY: year-over-year

Department	FY 2021 (Q1-Q4) wRVUs	FY 2022 (Q1-Q4) wRVUs	% chg YoY	Impact to physician generated wRVUs
Community Practice Primary care	1,096	3,485	218%	None
Radiological Sciences	3,044	7,096	133%	None
Medicine	20,319	43,322	113%	None
Plastic Surgery	617	1312	113%	None
General Surgery	13,586	23,801	70%	None
Otolaryngology	3328	5,071	52%	None
Orthopaedic Surgery	2,867	3,870	35%	None
Urology	6548	8624	32%	None
Psychiatry	3295	4203	27%	None

**Figure 2:**
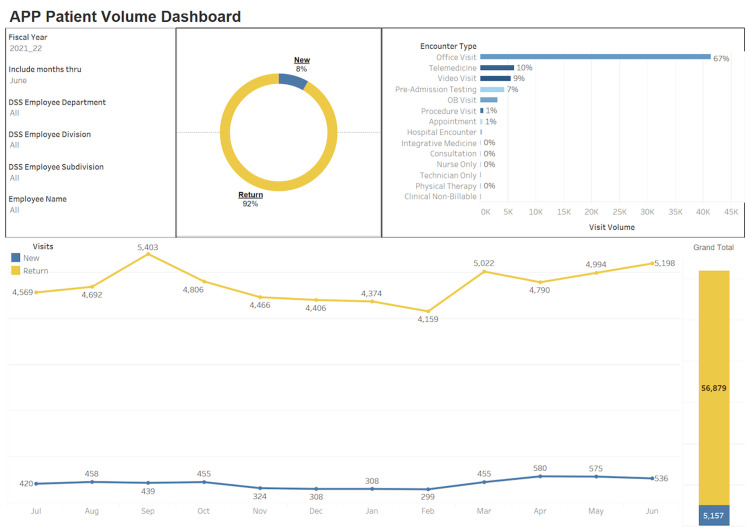
APP Ambulatory Visit Volume Dashboard APP: advanced practice provider, DSS: decision support services

With reference to APP ambulatory clinical effort, there was a 45% increase in the number of APP completed visits with 92% being return visits and 8% being new patient visits. Please refer to the APP financial and clinical dashboard (Figure 2). 

Additionally, shifting of empaneled established patients to PAs and NPs provided faculty physicians with greater accessibility and incremental capacity for new patient visits. For example, the overall “New to department visits” for established faculty increased by 13.1% in fiscal year (FY) 22. But when factoring overall growth with new faculty full time equivalents (FTEs), “New to department visits” increased by 22.8% in FY 22. Additionally, practice capacity utilization metrics were closely monitored to avoid cannibalizing appointments from physician faculty. Furthermore, an increase in PA and NP productivity did not impede ramp-up expectations for new faculty hires or delay programmatic expansion efforts.

Patient satisfaction

Regarding patient perceptions of satisfaction with APP care across ambulatory sites, we used results from surveys conducted by National Research Corporation (NCR health). For FY 22, our ambulatory APP cohort received a net promotor score (NPS) of 85.7 for recommend providers office, which shows that approximately 90% of patients are loyal and satisfied with the care they received and would continue to see that provider, refer others, and would speak highly of our organization. Additionally, our ambulatory APPs received 88.2% for “rating of provider”, which supports the great care and trust they have with their APP provider (Table 3).

**Table 3:** Patient Satisfaction Data

Question	YTD Score	Last 3 Months	Last month	n-size	Score	Benchmark	Gap
Rating of provider	88.3	87.7	89.3	1009	88.2	87.7	0.5
Recommend Office	85.7	84.9	86.7	988	85.7	78.3	7.4

APP engagement survey

In a 2022 engagement survey, 58 respondents replied to a series of questions. PAs and NPs were asked how often they practice at the top-of-license and the majority of respondents (78.0%) reported working either “most of the time” or “always” at the top-of-license while 19.0% felt underutilized in their current clinical role. When asked whether clinical responsibilities are distributed evenly across your team, 63.6% of APPs reported “yes” while 36.4% said “no”. In probing whether staff members (i.e., registered nurses [RNs] licensed vocational nurses [LVNs], and medical assistants [MAs]) have a good understanding of the PA and NP role in the practice setting, 82.8% of respondents reported “yes” and 17.2% said “no’.

## Discussion

Although most current published research has investigated PA/NP clinical productivity and cost-effectiveness in a single medical specialty such as primary care, oncology, plastic surgery, and emergency medicine, this rare study examined the impact of implementing PA/NP autonomous practice models on ambulatory capacity, patient satisfaction, and provider productivity (wRVU analysis) across multiple clinical specialties. The adoption of ambulatory workload and patient facing expectations at our organization led to significant improvements in APP direct billable revenue, ambulatory visits, and other KPIs without adversely impacting ambulatory practice dynamics or physician productivity. Furthermore, APPs felt empowered to practice at the fullest extent of their training, and licensure in the independent visit model (IVM). This finding corroborates previous research, which found that APPs are very satisfied with IVMs [10]. 

While our study demonstrates improvements in operational and financial performance, we would be remiss, however, if we did not acknowledge that PAs and NPs often provide additional benefits such as committee work, care coordination, peer-to-peer prior authorization calls, onboarding of new employees, and ancillary services that often do not generate measurable revenue. A previous time motion study found that slightly less than one-third of the work completed by NPs and PAs does not generate RVUs [11]. Consequently, in a self-reported time study of two weeks’ duration at a large academic medical center, researchers found that APPs were spending approximately 36% of their time in direct patient care activities (billable plus bundled services), indicating opportunities to improve APP optimization [12]. 

Nevertheless, in today's market-driven environment, AMCs and hospital systems are seeking to leverage APPs to help boost revenue and productivity by having physicians focus their efforts on managing the increasing complexity of patients. In an era of increasing patient volumes, rising healthcare costs, projected physician shortages, and limited availability of in-house trainees, research consistency demonstrates the value of employing APPs at AMCs [13-15].

Our study had several limitations. First, this is a retrospective review and data was collected from one single academic medical center and further work is needed to determine the applicability of these findings at other AMCs and hospital systems. Second, differences in organizational and workplace cultures, care models, and governance structures at other AMCs may limit the generalizability of our findings. Third, we did not investigate whether PAs/NPs saw more patients per unit time than faculty physicians within the same specialty. Fourth, we did not examine the overall impact of independent practice sessions on clinical outcomes and job satisfaction of advanced practice staff and faculty as that was beyond the scope of the current study. 

## Conclusions

This quality improvement study demonstrates that implementing independent APP ambulatory practice sessions improves clinical capacity, patient satisfaction, department efficiency, workflow optimization, and downstream revenue opportunities. For those AMCs and hospital systems interested in replicating our process, there were several key takeaways that should be considered. First, create workgroups by clinical department comprised of APPs, physicians, practice managers, department administrators and other key stakeholders to review current state and consider strategies to optimize APP workflows and patient care goals. Second, define ambulatory practice expectations per clinical fraction full-time equivalent (cFTE) and achievable implementation roadmap. Third, evaluate change readiness through qualitative and/or quantitative assessments. Fourth, align APP ambulatory practice expectations with your organization’s recruitment plans, expected growth, and strategic priorities. For example, hiring of new faculty may lead a department to reallocate clinic space and patient visits from the APP to the newly hired faculty member. Fifth, identify provider champions in each specialty to assist with buy-in and understanding of performance expectations. Sixth, identify sufficient clinical space to support the number of anticipated PA and NP sessions based on template design. Also, calculate additional FTEs needed to provide ambulatory ancillary support for fully independent APP clinic templates. It is essential to develop a plan to offload below-license tasks to other members of the care team to ensure APPs have sufficient time to focus on patient facing activities. Anticipate APP salary shortfall due to ramp-up expectations and identify strategies to cover any projected salary deficit. Lastly, track and monitor APP productivity metrics through a centralized dashboard that is internally and externally validated. 
